# Annual acknowledgement of manuscript reviewers

**DOI:** 10.1186/2043-9113-3-5

**Published:** 2013-03-22

**Authors:** Xiangdong Wang

**Affiliations:** 1 Fudan University Zhongshan Hospital, Zhongshan, China

## Abstract

**Contributing reviewers:**

The editors of *Journal of Clinical Bioinformatics* would like to thank all our reviewers who have contributed to the journal in Volume 2 (2012).

## 

Avner Bar-Hen

France

Christian Baumgartner

Austria

Phei Lang Chang

Taiwan

Li Chen

United States of America

Luonan Chen

China

Yu-Huei Cheng

Taiwan

Claudia dos Santos

Canada

Alastair Droop

United Kingdom

Hariklia Eleftherohorinou

United Kingdom

Eva Enns

United States of America

Maria Eriksson

Sweden

Roland Foisner

Austria

Richard Gronostajski

United States of America

Xiaowei Guan

United States of America

Tao Han

United States of America

Md Rafiul Hassan

Australia

Dieter Heermann

Germany

John Hutton

United States of America

Kunlin Jin

United States of America

Anders Johnsen

Denmark

Radha Krishna Murthy Karuturi

Singapore

Lukas Kenner

Austria

Ju Han Kim

South Korea

Petra Knaup

Germany

Charles Kooperberg

United States of America

Hans Kreipe

Germany

Hane Lee

United States of America

Lance Liotta

United States of America

Long Lu

United States of America

Avi Ma'ayan

United States of America

Leah Mechanic

United States of America

Pablo Moscato

Australia

Michael Netzer

Austria

Casey Overby

United States of America

Nitin Patel

United States of America

Jeroen Pennings

Netherlands

Joshua Plavnick

United States of America

Helmut Popper

Austria

Cristin Print

New Zealand

Julia Sequi

Spain

Ambuj Singh

United States of America

K. Stephen Suh

United States of America

Juan Antonio Vizcaino

United Kingdom

Xiangdong Wang

Sweden

Christopher Woelk

United States of America

Junming Yue

United States of America

